# Effect of private versus emergency medical systems transportation in trauma patients in a mostly physician based system- a retrospective multicenter study based on the TraumaRegister DGU®

**DOI:** 10.1186/s13049-016-0252-1

**Published:** 2016-04-27

**Authors:** Stephan Huber, Moritz Crönlein, Francesca von Matthey, Marc Hanschen, Fritz Seidl, Chlodwig Kirchhoff, Peter Biberthaler, Rolf Lefering, Stefan Huber-Wagner, and the TraumaRegister DGU

**Affiliations:** Department of Trauma Surgery, Klinikum rechts der Isar, Technical University of Munich - TUM, Ismaninger Str. 22, D-81675 Munich, Germany; IFOM – Institute for Research in Operative Medicine, University Witten/Herdecke, Faculty of Health, Ostmerheimer Str. 200, D-51109 Cologne, Germany

**Keywords:** Polytrauma, Private transport, Emergency services, Emergency department

## Abstract

**Background:**

The effects of private transportation (PT) to definitive trauma care in comparison to transportation using Emergency Medical Services (EMS) have so far been addressed by a few studies, with some of them finding a beneficial effect on survival. The aim of the current study was to investigate epidemiology, pre- and in-hospital times as well as outcomes in patients after PT as compared to EMS recorded in the TraumaRegister DGU®.

**Methods:**

All patients in the database of the TraumaRegister DGU® (TR-DGU) from participating European trauma centers treated in 2009 to 2013 with available data on the mode of transportation, ISS ≥ 4 and ICU treatment were included in the study. Epidemiological data, pre- and in-hospital times were analysed. Outcomes were analysed after adjustment for RISC-II scores.

**Results:**

76,512 patients were included in the study, of which 1,085 (1.4 %) were private transports. Distribution of ages and trauma mechanisms showed a markedly different pattern following PT, with more children < 15 years treated following PT (3.3 % EMS vs. 9.6 for PT) and more elderly patients of 65 years or older (26.6 vs 32.4 %). Private transportation to trauma care was by far more frequent in Level 2 and 3 hospitals (41.2 % in EMS group vs 73.7 %). Median pre-hospital times were also reduced following PT (59 min for EMS vs. 46 for PT). In-hospital time in the trauma room (66 for EMS vs. 103 min for PT) and time to diagnostics were prolonged following PT. Outcome analysis after adjustment for RISC-II scores showed a survival benefit of PT over EMS transport (SMR for EMS 1.07 95 % CI 1.05–1.09; for PT 0.85 95 % CI 0.62–1.08).

**Discussion:**

The current study shows a distinct pattern concerning epidemiology and mechanism of injury following PT. PT accelerates the median pre-hospital times, but prolongs time to diagnostic measures and time in the trauma room.

**Conclusions:**

In this distinct collective, PT seemed to lead to a small benefit in terms of mortality, which may reflect pre-hospital times, pre-hospital interventions or other confounders.

## Background

Severely injured patients usually are transported to the treating hospital by means of the Emergency Medical Systems (EMS) in most industrialized countries. However, a small proportion of patients nevertheless refers themselves to specialized trauma care using private transportation for various reasons.

There has been a firmly established paradigm that severely injured patients benefit from early on-scene stabilization before transport to a hospital for definitive trauma care [1], however this paradigm has been challenged. Recent reports often favor short on-scene times with as little stabilization as necessary and rapid transfer to definitive care [2–4].

Fast transportation, for example by Helicopter Emergency Medical Systems, has been shown to be beneficial for outcomes [5, 6].

Private transportation (PT) to definitive care is in a way the most radical form of the latter paradigm, with expected quick transport times to definitive care and no means of on-scene stabilization.

However, in most systems of trauma care in industrialized countries, emergency department personnel and first-treating physicians usually rely on early announcement of severely injured patients to accelerate the in-hospital workflow. Self-referral challenges these habits as no notice is given in advance, thus potentially prolonging the in-hospital workflow.

There are previous reports on this topic, which analyzed the outcome of private transportation to definitive care. These reports cover a very heterogeneous spectrum of settings and geographic locations.

The effect of PT vs. use of EMS has been previously addressed for gunshot wounds, finding that victims of gunshot wounds have preferable outcomes when transported to a trauma center by private vehicles or police vehicles as compared to transport via EMS [7, 8]. The benefit was attributed to faster transport to definitive care, but prehospital times were not recorded. Another study found a lower rate of unexpected deaths following transport by police after blunt trauma as compared to EMS transport with otherwise comparable overall results regardless of penetrating or blunt trauma [8].

Mixed collectives of blunt and penetrating trauma have also been reported from the United States with some studies showing a benefit of individual transport on outcome, even when adjusted for indicators of injury severity such as the ISS [9]. However, no beneficial effect was found in a prospective study, which identified faster times to definitive care, but no significant benefit on survival and morbidity, potentially due to its small study size [10].

Of note, the German EMS system is based on a ‘rendezvous model’ [11]. In case of suspected severe injury, an emergency physician is dispatched to the scene, where first treatment is carried out by paramedics in the meantime. Physicians are dispatched by the rescue center following certain keywords such as “multiple injuries” or “altered consciousness”.

Thus, German Emergency Medical Services vary from the more widespread paramedic system.

Given the sparse evidence considering the effect of PT, especially from systems using a rendezvous model like Germany and many previously unaddressed or only sparsely addressed factors such as pre-hospital and in-hospital times, we sought to investigate these factors in the database of the TraumaRegister DGU®.

The aim of the current study thus was to investigate the incidence, epidemiology and in-hospital times of private transport to definitive care in a European study collective and to investigate the outcomes compared to transport using EMS.

## Methods

### Study design

This study was designed as a retrospective cohort study on data of trauma victims recorded in the TraumaRegister DGU®. The observation period was from 2009, when the option to enter private transport as a means of transportation was introduced to documentation in the TraumaRegister DGU® to the most recent available data set from 2013. Further details on the TraumaRegister DGU® and participating hospitals can be found under the paragraph data collection.

We identified patients who were classified as “self-referral” in the database of the TraumaRegister DGU® and compared them to patients who were transported to the hospital by any means of Emergency Medical Systems (EMS).

The German EMS system is based on a ‘rendezvous model’. In case of suspected severe injury, an emergency physician is dispatched to the scene, where first treatment is carried out by paramedics in the meantime. Physicians are dispatched by the rescue center following certain keywords such as “multiple injuries” or “altered consciousness”. Furthermore, German HEMS are generally accompanied with an emergency physician.

### Inclusion criteria in the study

Inclusion criteria were treatment via the trauma room, ISS ≥ 4, ICU treatment and available data on the form of transportation to the hospital; only patients from European trauma centers were included and secondary referrals were excluded from the study. These inclusion criteria are general inclusion criteria of the TraumaRegister DGU®.

For outcome analysis, patients who were transferred early (where data on outcome is not available) and whose documentation on patient age was missing could not be included.

### Data collection

The TraumaRegister DGU® of the German Trauma Society (Deutsche Gesellschaft für Unfallchirurgie, DGU) was founded in 1993. The aim of this multi-centre database is an anonymous and standardized documentation of severely injured patients.

Data are collected prospectively in four consecutive time phases from the site of the accident until discharge from hospital: A) Pre-hospital phase, B) Emergency room and initial surgery, C) Intensive care unit and D) Discharge. Documentation includes detailed information on demographics, injury pattern, comorbidities, pre- and in-hospital management, course on intensive care unit, relevant laboratory findings including data on transfusion and outcome of each individual. The inclusion criterion is admission to hospital via emergency room with subsequent ICU/ICM care or reaching the hospital with vital signs and death before admission to ICU.

The infrastructure for documentation, data management, and data analysis is provided by AUC - Academy for Trauma Surgery (AUC - Akademie der Unfallchirurgie GmbH), a company affiliated to the German Trauma Society. The scientific leadership is provided by the Committee on Emergency Medicine, Intensive Care and Trauma Management (Sektion NIS) of the German Trauma Society. The participating hospitals submit their data anonymously into a central database via a web-based application. Scientific data analysis is approved according to a peer review procedure established by Sektion NIS. The participating hospitals are primarily located in Germany (90 %), but a rising number of hospitals of other countries contribute data as well (at the moment from Austria, Belgium, China, Finland, Luxembourg, Slovenia, Switzerland, The Netherlands, and the United Arab Emirates). Currently, approx. 25,000 cases from more than 600 hospitals are entered into the database per year.

Participation in TraumaRegister DGU® is voluntary. For hospitals associated with TraumaNetzwerk DGU®, however, the entry of at least a basic data set is obligatory for reasons of quality assurance.

The criteria for definition of hospitals as Level 1, 2 or 3 centres can be found in the the current version of the Whitebook Medical Care of the Severely Injured, 2nd revised and updated edition (http://www.dgu-online.de/fileadmin/published_content/5.Qualitaet_und_Sicherheit/PDF/2012_DGU_Whitebook_Medical_Care_2ndEdition.pdf).

The present study is in line with the publication guidelines of the TraumaRegister DGU® and registered as TR-DGU project ID 2014–061.

### Statistical analysis

The group of PT patients was compared to the group of patients brought to hospital via EMS. Metric variables were presented as mean with standard deviation (SD) and median, categorical variables were presented as percentages. Since the EMS group is rather large (75,000), and the PT group also consisted of about 1000 cases, formal statistical evaluation was avoided because even minor differences would formally become statistically significant. Since prognostic factors and outcome was different in the two groups, the observed mortality was compared to the prognosis based on the RISC II score within each group, which has recently been shown to be a predictive scoring system with good discrimination, precision, and calibration [12]. The quotient of both rates (observed divided by expected mortality rate) is known as standardized mortality ratio (SMR). SMR values are presented together with their 95 % confidence interval (95 % CI) which was based on the 95 % CI of the observed mortality. If the value of 1 is contained in the 95 % CI of the SMR then the outcome is within the expected range, and deviations might be explained by chance (using a 5 % error rate). All statistical analyses were performed using SPSS statistical software (Version 21, IBM Inc., Armonk, NY, USA).

## Results

### Patient demographics

**Fig. 1:**
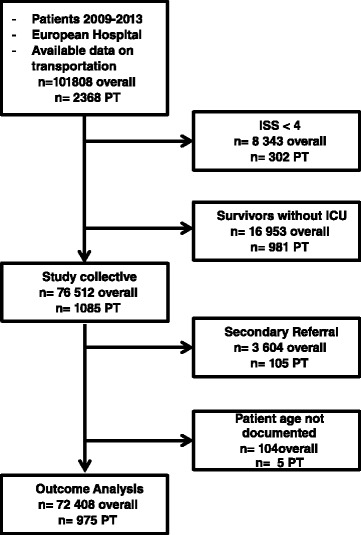
shows the study algorithm of patients included in the study

Within the observation period from 2009 to 2013, more than 101,000 patients (2,368 of which came to trauma care with private transport (PT)) documented in the TraumaRegister DGU® (TR-DGU) met the primary inclusion criteria. From these, 8,343 (PT: 302) patients had an ISS < 4 and 16,953 (PT: 981) patients were survivors who were not treated in intensive care; according to general exclusion criteria of the TraumaRegister DGU®, these were also excluded from the study. Thus, 76,512 patients were included in the study, of which 1,085 (1.4 %) were private transports (see Fig. 1)

The demographics were initially comparable in the two groups with a mean age of 48.2 (EMS) vs. 49.4 (PT) years; 71.0 % vs. 74.6 % of the patients were male. Blunt trauma accounted for the vast majority in both groups (95.3 % EMS vs. 94.7 % PT). The mean ISS however was lower in the PT group as compared to the EMS group (14.4 ± 8.7 vs 20.1 ± .13.0, *p* < 0.0001).

Detailed information concerning demographics in the respective groups is depicted in Table 1.

**Table 1:** Demographics of study patients

	Age	Injury Severity (ISS)	Blood pressure (mmHg)	Ventilator Days (days)	ICU Stay (days)	Hospital Stay (days)		Sex (%)
EMS								
Mean	48.24	20.1	122.6	3.42	6.9	17.8		
Standard Deviation	22.0	13.0	39.4	8.1	10.8	19.3	Male	71.0
Median	48	17	128	0	3	13	Female	29.0
*n*=	75097	75427	75427	74775	75426	75389		
PT								
Mean	49.4	14.4	113.5	0.8	3.5	11.4		
Standard Deviation	24.3	8.7	53.9	3.6	5.2	11.1	Male	74.6
Median	51	13	130	0	2	9	Female	25.4
*n*=	1085	1085	1085	1077	1085	1085		

**Fig. 2:**
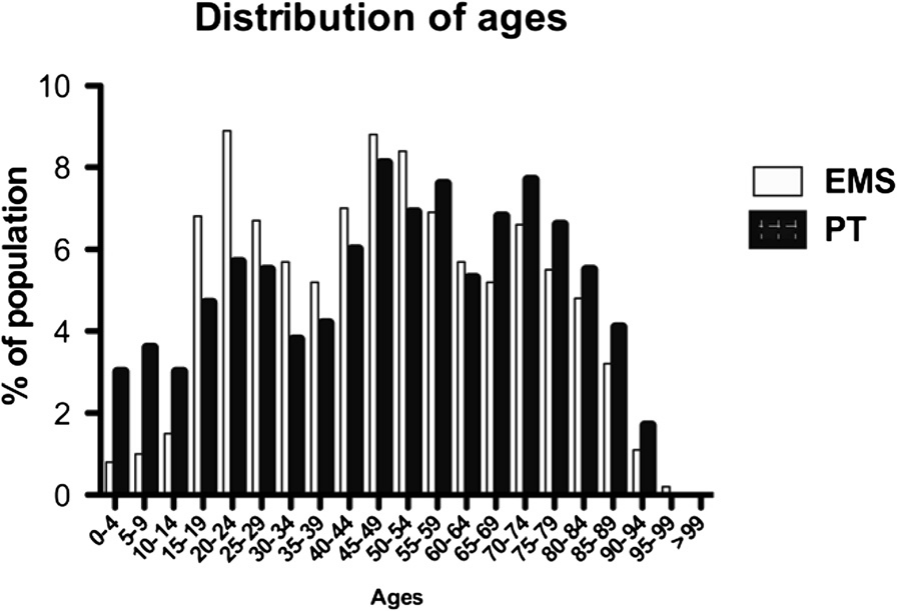
shows the distribution of ages subdivided in decades of the two study groups

The distribution of ages however showed that children < 14 years and older people of >65 years were represented to a higher degree in the private transportation group (Fig. 2).

### Mechanism of injury

All forms of Road Traffic Crashes (RTCs) with the exception of cycling accidents were rarer in the Private Transportation group as compared to the Emergency Medical Services Group. Overall, RTCs made up only 21.5 % of mechanisms of injury as compared to 54.1 % in the EMS group.

Minor falls (<3 m) were by far more common in the PT group (43.5 % EMS vs. 18.0 % PT).

All forms of physical violence as a whole were rare in both groups; however while gunshot wounds were equally infrequent in both groups (0.6 % EMS vs. 0.5 % PT), assault and stab wounds were more frequent in patients after PT (assault: 3.0 % EMS vs. 6.2 % PT; stab wounds: 1.7 % EMS vs. 3.7 % PT). For further details see Table 2.

**Table 2:** Patterns of injury, distribution of types of EMS transport, level of trauma center in the study population

	EMS (%)	PT (%)
Car passenger	24.1	2.5
Motorcyclist	13.3	5.3
Cyclist	8.3	11.1
Pedestrian	7.2	1.8
Other Road Traffic Crashes	1.2	0.8
Fall >3 m	17.0	14.2
Fall <3 m	18.0	43.5
Assault	3.0	6.2
Gunshot	0.6	0.5
Stab wounds	1.7	3.7
Others	5.6	10.4
Blunt trauma	95.3	94.7
Penetrating trauma	4.7	5.3
Mean ISS (points)	20.1	14.4

### Form of transport

Forms of transportation in the EMS group were further classified. The majority of patients were transferred at ground level in the presence of an emergency physician (67.8 %). Helicopter transport (24.1 %) and ground level transportation without the presence of an emergency physician (8.2 %) were less frequent (see Table 3.

**Table 3:** Patterns of injury, distribution of types of EMS transport, level of trauma center in the study population

Distribution of EMS	%
Helicopter Transport	24.1
Ground Transport with Emergency Physician	67.8
Ground Transport without Emergency Physician	8.2

### Types of hospital in the study population

We further analyzed to which types of hospitals trauma patients are transferred via PT. While over half of the patients were transferred to Level 1 centers when EMS was involved (58.8 %), only about a quarter of patients (26.4 %) presented to Level 1 centers on their own initiative.

Level 2 and 3 hospitals represented a by far larger proportion of patients after PT as compared to EMS-transferred patients (Level 2: 33.3 % EMS vs. 46.2 % PT; Level 3: 7.9 % EMS vs. 27.5 % PT; see Table 4).

**Table 4:** Patterns of injury, distribution of types of EMS transport, level of trauma center in the study population

	EMS		PT	
	n	%	n	%
Level 1-Center	44378	58.8	286	26.4
Level 2-Center	25122	33.3	501	46.2
Level 3-Center	5927	7.9	298	27.5

### Times to first treatment

The mean time to first in-hospital treatment is very similar in EMS and PT groups (62.7 min EMS vs. 62.3 min PT).

However, the difference in median times (59 min EMS vs. 46 min PT) suggests that indeed many patients arrive faster at the hospital using PT than expected.

**Fig. 3:**
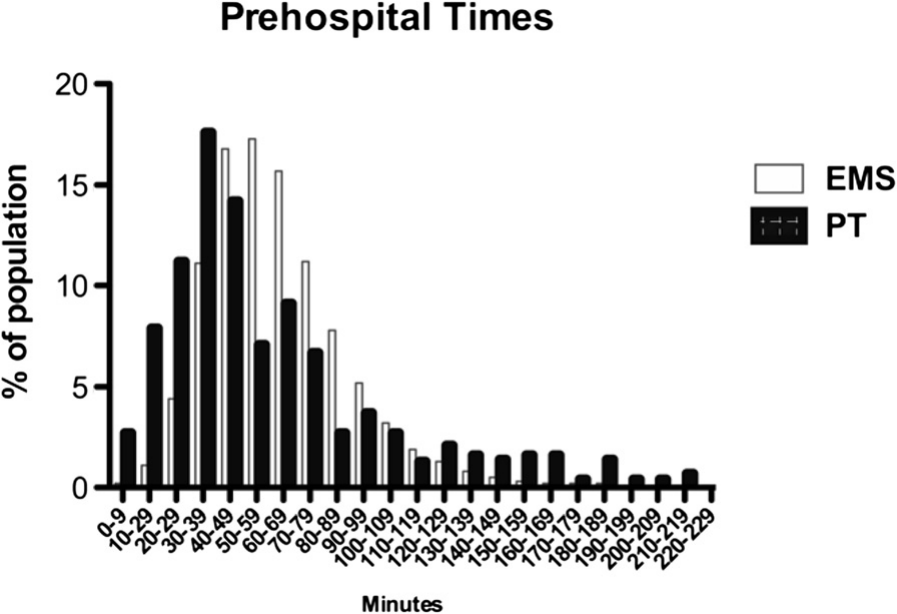
shows the distribution times to treatment of the two study groups subdivided in ten minute intervals

As can be seen in Fig. 3, late transport to definitive care is also more common in the PT group.

### Treatment times in the study population

In those cases where it was documented, treatment time in the trauma room until transfer to operation or ICU was drastically prolonged (65.6 min EMS vs. 102.5 min PT, see Table 5).

**Table 5:** In-hospital treatment times of PT vs. EMS groups

	Time to sonography (min)	Time to X-Ray (min)	Time to CT-Scan (min)	Time in trauma room (min)
EMS				
Mean	6.0	14.5	23.3	65.6
Standard Deviation	8.1	18.1	17.8	44.4
Median	4	9	20	53
*n*=	54,678	30,614	63,190	33,068
Private				
Mean	14.7	35.6	43.4	102.52
Standard Deviation	17.6	31	34.1	66.7
Median	9	26	32	80
*n*=	576	448	612	297

Furthermore, the time to first diagnostics was drastically prolonged following private transport. Sonography (FAST) was performed after an average time of 6 min in the EMS group, while in the PT group, FAST was performed 14.7 min after arrival. The delay in diagnostic procedures was even more pronounced for X-Ray (14.5 min EMS vs. 35.6 min PT) and CT (23.3 min EMS vs. 43.4 min PT).

### Outcome analysis

The observed mortality after transportation using Emergency Medical Systems was 13.2 % (95 % CI 13.0–13.5 %) as compared to 5.0 % (95 % CI 3.7–6.4 %) after Private Transportation.

After adjustment for RISC-II scores, the expected mortality was 12.4 % in the EMS and 5.9 % in the PT group. This difference is indicative of the differences in injury severity.

The Standardized Mortality Ratio was 1.07 after EMS transport (95 % CI 1.05–1.09) and 0.85 (95 % CI 0.62-1.08) after PT, see Table 6.

**Table 6:** Outcomes after PT vs. EMS

	n	%	upper 95 % CI	lower 95 % CI	expected	SMR	95 % for SMR
EMS	9,464	13.2	13	13.5	12.4	1.07	1.05 – 1.09
PT	49	5	3.7	6.4	5.9	0.85	0.62 – 1.08

## Discussion

Our retrospective, multicenter study addresses the comparative epidemiology, times and outcomes of Private Transportation versus the use of Emergency Medical Systems in all patients registered in TraumaRegister DGU® to definitive care during a study period of four years.

A retrospective, large-cohort analysis of PT versus EMS was investigated using the American College of Surgeons National Trauma Data Bank (NTDB), but with a focus on gunshot wounds. Favorable outcomes for Private Transportation were found in the study [9]. There is however only little comparability between this study and our study, as our study collective consists almost exclusively of patients suffering from blunt trauma with less than 1 % gunshot injuries in the population.

A local, retrospective cohort study of trauma patients in Philadelphia, USA, a single urban metropolitan setting, investigated police transport in comparison to EMS transport with regards to survival [8]. Adjusted for injury severity, no overall survival benefit was found after police transport, but patients in subgroups with critical penetrating injuries (gunshot, stab wounds) and critically injured patients were more likely to survive following police transport.

In another American retrospective single-center study with a mixed collective of blunt and penetrating trauma, a beneficial effect of PT has been identified in all patients meeting criteria for major trauma presenting to a single, urban Level-1-center [13]. This study however also shows limited comparability to our current study. Only little patients presented to Level-1-centers in our study, also suggesting a largely rural population in our study.

A prospective single-center study from the same center with a rather small study size of slightly over 100 patients could not confirm a significant effect of PT on survival, but showed that expectedly definitive care could be provided earlier to many critically injured patients, even though mean transfer time was similar [10].

A retrospective single center study from the Sultanate of Oman revealed a non-significant reduction in mortality after EMS transport; however only patients after road traffic crashes were included in this study [14].

Given the sparse nature of previous studies and their heterogeneity, our study offers novelty value regarding various aspects.

First of all, mechanisms of injury and the epidemiology in studies from the United States and Oman are different to those in Europe, as our study highlights. Some studies from the United States focus on gunshot wounds, a mechanism of injury nearly unrepresented in our study with less than 1 % of injuries caused by firearms.

RTCs, especially in car passengers are the focus of the study from the Sultanate of Oman [14]. This is in contrast to our study collective, where RTCs overall represent less than a quarter of injuries in the PT group and car passengers are only a small subgroup of these. Also, since less than 8 % of patients had been admitted to ICU in that study, the study collective is totally different from ours where admission to ICU or in-hospital death prior to admission is mandatory for inclusion in the study.

Only one of the aforementioned studies investigated pre-hospital time in comparison between PT and EMS [10]. No significant difference between the two groups could be detected, however many critically injured patients were in definitive care comparably early. Similar results could be obtained in our study. The mean time to admission was very similar in our study between the two groups, but the distribution of times to treatment was different. As suggested by the pronounced difference in the median pre-hospital time and standard deviation, more patients arrived in definitive care in the first 40 min after trauma following PT confirming the previous study. However, substantially more late arrivers were admitted over 120 min after trauma, suggesting many initially underestimated injuries. In general, both groups – even the EMS group –had a rather fast mean pre-hospital time of 62 min as compared to other collectives from the TraumaRegister DGU® [15, 16].

Our study is the first to report on the effect and epidemiology of PT as compared to a physician-based EMS system. In contrast to previous studies on PT, which were conducted in countries with a paramedic system where Advanced Life Support is provided (USA, Oman) [7–10, 14], the German EMS system relies on emergency physicians, which are dispatched to the scene following all suspected cases of life-threatening trauma or illness. This is reflected by the fact that a physician was present in over 90 % of EMS transports in our study.

Comparative studies have shown that pre-hospital times after trauma are prolonged in physician-based countries. German pre-hospital times were found to be prolonged in comparison to those in the USA and more patients received endotracheal intubation on-scene [11].

For the first time, our study shows that the time in the trauma room is substantially prolonged following PT to trauma care. The reasons have not been addressed previously and remain speculative, of course, but some factors can be addressed.

First of all, many time-consuming procedures such as endotracheal intubation or chest tube placement are performed on-scene after physician-based EMS treatment, as a recent analysis from the TraumaRegister DGU® shows. Assuming some of these procedures have to be performed in the trauma room explains some of the delay [16].

Another recent analysis of the TraumaRegister DGU® showed that indeed the overall time from RTC to end of trauma room treatment was very hardly affected by invasive procedures on-scene [17].

The arrival of a patient with suspected severe injury by EMS is usually pre-announced in European countries and a team assembled in the trauma room, including specialists for Surgery, Anesthesiology and Radiology. Given the delay in diagnostic procedures, the lack of announcement may lead to a delay in alarming the team, as early trauma team activation leads to accelerated diagnostic measures [18]. In contrast, EMS transport may increase vigilance upon in-hospital treatment and thus accelerate treatment. This delay however does not appear to lead to inferior outcomes with regards to mortality.

Outcomes after PT versus EMS transportation have been mixed in reports from Western countries [7–10, 14]. None of the two modes of transport has so far been conclusively proven to be superior in terms of mortality, even though some prior studies have found benefits after PT or police transport. This effect has been described most often in patients suffering from gunshot wounds. The most obvious reason would of course be reduced pre-hospital time, but no study has so far shown that indeed patients arrive earlier in definitive care after PT. While our study shows no benefit concerning mean pre-hospital time, there was a substantial difference in the median time.

The limitations of the study arise firstly from its methodology. The study is strictly retrospective. Furthermore, as mentioned above, some potential biases have to be addressed in the interpretation of study results. Firstly, there is a potential selection bias given that patients in the PT group had a very different injury pattern and more simple falls. Furthermore, patients who might be stabilized by EMS providers on-scene arrive at the hospital despite adverse prognostics, whereas these patients are by nature absent in the PT group. Secondly, the two study groups of course show different mechanisms of trauma, thus reducing comparability. Thirdly, patients in the PT group referred themselves to different types of hospitals as EMS providers would refer patients to, leading to a stronger representation of Level 2 and Level 3 hospitals. A recent analysis suggested the volume of treated trauma patients as an independent prognostic factor not yet adjusted for in this study, thus potentially increasing the expected mortality in the PT group [19].

Furthermore, the study collectives of PT and EMS groups are very different in their properties and injury severity and the PT group is drastically smaller, thus introducing another potential bias.

Another limitation of the TraumaRegister DGU® is based on the fact that only a subgroup of hospitals participates in the extended documentation with a larger data set. This leads to a smaller study collective in the analysis of in-hospital times, which are documented only on a volunteer basis by participating hospitals. The sub-collectives may of course not be entirely representative for the entire study population and constitute a reporting bias.

## Conclusions

Our study shows a distinct pattern concerning epidemiology and mechanism of injury following PT that is significantly different from patients after EMS transport and also from previous study collectives from other systems of trauma care. PT accelerates the median pre-hospital times, but prolongs time to diagnostic measures and time in the trauma room. In this distinct collective, PT seemed to lead to a small benefit in terms of mortality, which may reflect pre-hospital times, pre-hospital interventions or other confounders.
